# Glucagon-Like Peptide-1 Analog Exendin-4 Ameliorates Cocaine-Mediated Behavior by Inhibiting Toll-Like Receptor 4 Signaling in Mice

**DOI:** 10.3389/fphar.2021.694476

**Published:** 2021-07-19

**Authors:** Changliang Zhu, Hong Tao, Shikuo Rong, Lifei Xiao, Xinxiao Li, Shucai Jiang, Baorui Guo, Lei Wang, Jiangwei Ding, Caibing Gao, Haigang Chang, Tao Sun, Feng Wang

**Affiliations:** ^1^ Department of Neurosurgery, General Hospital of Ningxia Medical University, Yinchuan, China; ^2^ Ningxia Key Laboratory of Cerebro Cranial Disease, Incubation Base of National Key Laboratory, Ningxia Medical University, Yinchuan, China; ^3^ Department of Neurosurgery, The First Affiliated Hospital of Nanchang University, Nanchang, China; ^4^ Department of General Surgery, Chengdu Second Hospital, Chendu, China; ^5^ Department of Neurosurgery, The First Affiliated Hospital of Zhejiang University School of Medicine, Hangzhou, China

**Keywords:** cocaine, condition place preference, extinction, reinstatement, exendin-4, neuroinflammation, toll-like receptor 4

## Abstract

Exendin-4 (Ex4), a long-lasting glucagon-like peptide-1 analog, was reported to exert favourable actions on inhibiting cocaine-associated rewarding and reinforcing effects of drug in animal models of addiction. However, the therapeutic potential of different dose of GLP-1 receptor agonist Ex4 in different behavioral paradigms and the underlying pharmacological mechanisms of action are incompletely understood. Herein, we firstly investigated the effects of Ex4 on cocaine-induced condition place preference (CPP) as well as extinction and reinstatement in male C57BL/6J mice. Additionally, we sought to elucidate the underlying pharmacological mechanism of these actions of Ex4. The paradigm of cocaine-induced CPP was established using 20 mg/kg cocaine or saline alternately during conditioning, while the reinstatement paradigm was modeled using 10 mg/kg cocaine on the reinstatement day. Different dose of Ex4 was administrated intraperitoneally either during conditioning or during extinction state or only on the test day. To elucidate the molecular mechanism underlying the potential effects of Ex4 on maladaptive behaviors of cocaine, the TLR4-related inflammation within the hippocampus was observed by immunofluorescence staining, and the expression levels of toll-like receptor 4 (TLR4), tumor necrosis factor (TNF)-α, and interleukin (IL)-1β were detected by Western blotting. As a consequence, systemic administration of different dose of Ex4 was sufficient to inhibit the acquisition and expression of cocaine-induced CPP, facilitate the extinction of cocaine-associated reward and attenuate reinstatement of cocaine-induced behavior. Furthermore, Ex4 treatment diminished expression levels of TLR4, TNF-α, and IL-1β, which were up-regulated by cocaine exposure. Altogether, our results indicated that Ex4 effectively ameliorated cocaine-induced behaviors likely through neurobiological mechanisms partly attributable to the inhibition of TLR4, TNF-α and IL-1β in mice. Consequently, our findings improved our understanding of the efficacy of Ex4 for the amelioration of cocaine-induced behavior and suggested that Ex4 may be applied as a drug candidate for cocaine addiction.

## Introduction

Addiction to drugs is an enormous health issue worldwide; approximately 19 million people worldwide were addicted to cocaine in 2018, corresponding to 0.4% of the global population aged 15–64 years (Niaz et al., 2020). Emerging surveys indicate that the proportion of cocaine consumption is climbing (Hughes et al., 2016). Cocaine addiction is a chronic brain disease characterized by high incidence of relapse to compulsive behavior following detoxification, regardless of catastrophic consequence (Leshner, 1997). However, the efficacy of available opioid receptor antagonists and therapeutic options for the treatment of cocaine use disorder has been shown to be limited (Jerlhag, 2019). Thus, there is a clear need to identify novel therapeutic drugs for the treatment of cocaine use disorder.

Glucagon-like peptide-1 (GLP-1) is commonly referred to as an incretin hormone and satiation factor secreted predominantly by enteroendocrine Lcells and preproglucagon neurons (Coller and Hutchinson, 2012; Hutchinson and Watkins, 2014), which exerts metabolic effects on energy homeostasis and food intake in preclinical studies (Holst, 2007; Barrera et al., 2011; Turton et al., 2016). GLP-1 is rapidly degraded by dipeptidyl peptidase-IV which is abundantly expressed in the central nervous system (Crews et al., 2017). GLP-1 is supposed to activate GLP-1 receptor (GLP-1R), a G-protein coupled receptor, to stimulate the secretion of insulin and block the secretion of glucagon in a nutrient-dependent way to achieve the goal of regulating glucose homeostasis (Holst, 2007). GLP-1 receptor is highly expressed throughout the whole brain including the hippocampus (Merchenthaler et al., 1999; Lathe, 2001; Hamilton and Lscher, 2009; Holst et al., 2011; Isacson et al., 2011; Richards et al., 2014), which is traditionally thought to be the main brain region of memory and learning (Bohbot and Corkin, 2007) and involved in substance use disorder (Meyers et al., 2003; Meyers et al., 2006; Olmo et al., 2006). These data indicate that GLP-1 is capable of interacting with and modulating hippocampus, which is part of mesolimbic reward system and implicated in addiction-like behavior (Kenny, 2011; Grill and Hayes, 2012; Williams and Elmquist, 2012; Hayes and Schmidt, 2016; Jerlhag, 2019). Nevertheless, the clinical utility of GLP-1 is limited due to its relatively short half-life. In contrast to GLP-1, its analogue exendin4 (Ex4) possesses a longer half-life (Gallwitz, 2004), and easily crosses the blood-brain barrier (Holscher, 2010). Therefore, Ex4 are widely applied for the treatment of type II diabetes and obesity (Drucker and Nauck, 2006; Hamilton and Lscher, 2009; Lovshin and Drucker, 2009; Eng et al., 2014; Richards et al., 2014; Pi-Sunyer et al., 2015; Andersen et al., 2018). In addition, Ex4 has also caused considerable interest in recent years because of the possible actions on ameliorating the maladaptive behavior of cocaine such as cocaine-induced CPP as well as locomotor activity, and self-administration (Hernandez and Schmidt, 2019). However, few studies have explored the underlying effects of GLP-1R agonist Ex4 on extinction as well as reinstatement and its possible molecular mechanisms to date.

Recently, GLP-1R agonists have received increasing attention due to its neuroprotective (Holst et al., 2011) and anti-inflammatory effects (Hattori et al., 2010; Campbell and Drucker 2013; Lee and Jun 2016; Géa et al., 2020). For instance, GLP-1R agonists reportedly inhibited oxidative stress and inflammatory mediators in animal model of diabetes (Wu et al., 2011). In addition, Ex4 has been confirmed to be effective in reducing indices of inflammation, including TLR4, tumor necrosis factor (TNF)-α, and interleukin (IL)-1β levels (Ajay et al., 2011). Moreover, GLP-1R agonists have been examined to have a profound impacts on cell proliferation, growth, survival, and repair in preclinincal study of neurodegenerative diseases (Gault and Hölscher 2018). For instance, the favorable effects of Ex4 has also been confirmed in treating many neurodegenerative diseases characterized by chronic neuroinflammation, such as Alzheimer’s disease, Parkinson’s disease, Huntington diseases, and amyotrophic lateral sclerosis (Li et al., 2009; Martin et al., 2009; Li et al., 2010; Yazhou et al., 2012). Interestingly, the neuroinflammation pertinent to the aberrant expression TLR4, IL-1β and TNF-α has also been validated to be heavily implicated in chronic cocaine consumption in growing studies (Sekine et al., 2008; Cearley et al., 2011; Clark et al., 2013; Hutchinson and Watkins, 2014; Northcutt, et al., 2015; Lacagnina et al., 2016). However, it remain unclear whether the suppressive ability of GLP-1R agonists Ex4 to mitigate neuroinflammation could be repurposed to ameliorate the cocaine-induced behavior in mice.

Indeed, neuroinflammation has been widely considered to be involved in many central nervous system (CNS) diseases (Block and Hong, 2005; Chen et al., 2006; Tansey et al., 2007). In search for new therapeutic drug targets for substance abuse, the neuroinflammation has been found to play a significant role in neural adaptations after chronic drugs exposure, and the anti-inflammatory ingredients may represent a novel and effective therapeutic approach for behavioral treatment of substance use disorders (Kohno et al., 2019). The primary pathway related to neuroinflammatory processes is triggered by pattern recognition receptors (PRR), including endogenous danger signals (DAMPs), and microbes or invading pathogens (MAMPs/PAMPs), which recognized the possible threats such as pathogen invasion or tissue damage. Toll like receptors (TLRs), the most common PRR, are mainly disturbed on microglia (Hanamsagar et al., 2012), which was expressed throughout brain with highest levels in hippocampus, basal ganglia, and substantia nigra (Lawson et al., 1990). Indeed, TLR4-mediated neuroinflammation has been shown to play a pivotal role in alcohol (June et al., 2015), morphine (Hutchinson 2012), and cocaine-associated behaviors (Northcutt et al., 2015). Additionally, TLR4-mediated microglial activation promotes proinflammatory cytokine release including IL-1β and TNF-α (Brown et al., 2011) that were implicated in drug addiction (Correia et al., 2020). Therefore, neuroinflammation associated with TLR4, IL-1β, and TNF-α may play a pivotal role in substance abuse. For instance, these anti-inflammatory agents including naloxone Ibudilast and Minocycline have exerted promising actions in the treatment for substance use disorders (Garrido-Mesa et al., 2013; Ray et al., 2014; Northcutt, et al., 2015). Although these work have yielded intriguing outcomes, thus far, few studies has reported the effects of other anti-inflammatory agents on cocaine addiction (Correia et al., 2020). Consequently, we hypothesized that the favorable actions of GLP-1R agonists Ex4 as a novel anti-inflammatory agents on the amelioration of the maladaptive cocaine-induced behavior is exerted by suppressing TLR4-related neuroinflammation in mice.

To examine our hypothesis, we firstly evaluated the effects of Ex4 on modulating the acquisition and expression of cocaine-induced CPP as well as facilitating extinction and blocking reinstatement in animal model of cocaine-induced CPP. Finally, we attempted to explore the role of GLP-1R agonist Ex4 in reducing abnormal expression of TLR4, TNF-α, and IL-1β in hippocampus.

## Materials and Methods

### Animals

Adult male C57BL/6J mice (18–22 g body weight) were provided by the Experimental Animal Center of Ningxia Medical University (China). All animals were housed in a specific pathogen-free environment under a 12-h light/dark cycle with lights on from 07:00 AM to 07:00 PM, a constant room temperature (20–25°C) and ambient humidity (50–60%), and free access to water and rodent food. In order to reduce the psychological stress induced by intraperitoneal injections, each mouse was kept in the testing room for 30 min on consecutive days before the beginning of each experiment. The experiment was initiated after acclimation for one week. The animal study was approved by the Ningxia Key Laboratory of Cerebrocranial Disease. All experimental procedures involving mice were implemented according to national, regional, and local laws and regulations, in accordance with the guidelines established by the Animal Research Ethics Committee of Ningxia Medical University. We took great care to minimize animal suffering and the number of animals used in this study.

### Drugs and Antibodies

Cocaine-hydrochloride was obtained from China National Medicines Corporation Limited (Beijing, China) and was dissolved in sterile 0.9% physiological saline solution (0.9% NaCl) to a concentration of 4 mg/ml. The drug was freshly prepared for intraperitoneal (i.p.) administration at doses of 20 mg/kg body weight to induce the CPP paradigm or 10 mg/kg body weight to produce reinstatement of CPP. Saline was used as a vehicle solution. The GLP-1R agonist Ex4 was purchased from MedChemExpress (MCE, United States) and was diluted in 0.9% physiological saline solution at a concentration of 0.6 μg/ml. Ex4 was freshly prepared at 100.0, 30.0, 1.0, and 0.1 μg/kg body weight which has been shown to significantly decrease the rewarding effects of cocaine (Reddy et al., 2016.; Hernandez and Schmidt, 2019). Injections of Ex4 and cocaine-HCl were alternatively performed on the left or right side of the peritoneum. Ex4 was administered 1 h before repeated injections of cocaine (20 mg/kg, i.p.) or saline (5 ml/kg), and cocaine was administered immediately before the commencement of the trial. Although cocaine was injected only on the conditioning day, Ex4 was injected every day for 8 days. Primary antibodies, including anti-TLR4, anti-IL-1β, and anti-TNF-α antibodies, were purchased from Abcam (San Francisco, CA, United States). To evaluate the protein expression of TLR4 and components of pro-inflammatory (TNF-α and IL-1β) signaling pathways in the hippocampus, mice received 20 mg/kg of cocaine (Rodríguez-Arias et al., 2009; Araos et al., 2015). The cocaine-induced CPP paradigm was established to examine cocaine CPP extinction and reinstatement using 20 mg/kg (conditioning session) and 10 mg/kg (priming dose, reinstatement session). The dose for conditioning (20 mg/kg, i.p.) was chosen because it elicits strong CPP behavior (Meye et al., 2016; Ladrón de Guevara-Mirand et al., 2018). During the experiments, solutions and drugs were stored at 4°C, and antibodies were stored at −20°C.

### Experimental Design and Procedures

In our experiment, cocaine (20 mg/kg) or an equal volume of saline (5 ml/kg) were administered by intraperitoneal injection on alternate days. There were four different phases in our study: 1) preconditioning phase; 2) conditioning (training/acquisition); 3) extinction, conducted 8 days after the test 2, when the CPP score was determined, followed by drug administration; and 4) reinstatement, which occurred 24 h after the last extinction and was used to investigate reinstatement of cocaine-induced CPP by cocaine priming (10 mg/kg body weight) on the challenge day. The tests of CPP score were performed on days 2, 10, 14, 19, and 20. Mice were sacrificed, and hippocampus tissues were collected after behavioral examinations for western blot analyses (*n* = 5 mice/group) or immunofluorescence staining (*n* = 3 mice/group). During the experiment, none of the mice died.

### Conditioning Apparatus

The general procedure for the CPP was performed as previously described, with slight modifications (Meye et al., 2016). Briefly, the conditioning for CPP was performed using a shuttle apparatus consisting of two equally sized larger compartments (24 cm length × 14 cm width × 30 cm height) separated by a smaller compartment (7.0 cm length × 7.0 cm width × 30 cm height) with retractable guillotine doors; the larger compartment contained four black walls and a smooth black floor, the other compartment contained four white walls and a rough white floor covered with blue sandpaper. The two larger compartments had different visual and tactile cues. The gray central corridor contained twogray walls and a smooth floor leading into the corresponding compartment. The removable guillotine doors separated the three compartments to prevent animals from crossing the chambers and were raised on test days. Tracking of the mice in the apparatus was performed using an infrared video camera suspended approximately 1 m above the test arena. The overhead infrared camera and computer were used to record the real-time positions of the mice and their movement throughout the three compartments. The time spent in each chamber and total distance traveled were recorded using a computerized video tracking system (video behavior analysis software Smart 3.0; Panlab, Spain; supported by RWD Life Science Co., Ltd., China).

### Pre-Conditioning Stage (days 1–2)

On days 1–2, all mice were positioned in the middle area of the apparatus and permitted to freely explore all three compartments for 45 min prior to the commence of the experiment. Following habituation, the mice were again placed in the middle intersection of the chambers and allowed to explore freely for 20 min; their time spent in a white chamber (referred as non-preferred chamber) were recorded to set their basal preference. After completion of this test, biased mice with their time in any compartment more than 960 s or less than 240 s were excluded, and then these mice were randomly separated into four groups in response to the requirement of different experiments.

### Conditioning Stage (days 3–10)

On days 3–10, cocaine conditioning was conducted with cocaine (20 mg/kg, i.p.) or saline (5 ml/kg, i.p.) for 8 days, including four drug sessions and four saline sessions. Animals On days 3, 5, 7, and 9, mice were administered cocaine and then instantly confined to the designed cocaine- or saline-paired compartments by closing the removable guillotine doors for 45 min after treatment. The white compartment of the CPP cage in this trial was defined as the cocaine-paired compartment. On days 4, 6, 8, and 10, mice were confined to the opposite compartment for the same amount of time following saline injection. In this phase, all animals were conditioned once a day. Place preference testing was performed after an 8-days conditioning period. During this test, the mice were habituated in the center corridor and freely moved throughout the apparatus for 20 min. After the mice completed the test, mice were sacrificed for analysis by western blotting or immunofluorescence staining. The remaining mice were returned to their cages. The CPP score was calculated as the time spent in the cocaine-paired chamber during the test of 20 min. CPP was considered to be achieved when the CPP score was significantly higher on CPP tests than baseline preference.

### Condition Place Preference Extinction (days 11–19)

Following the acquisition and expression of cocaine-induced CPP, all animals were subjected to extinction training for eight days. During the extinction period, mice were not administered cocaine or Ex4 and were confined in the cocaine-paired compartment or saline-paired compartment for 45 min on alternate days. CPP testing of extinction was performed on day 19 to analyze the effects of treatment with Ex4 on extinction responses to cocaine-induced CPP after 8 days of extinction training. In this study, extinction was achieved when there was no significant difference in CPP scores compared to pretest. No drug treatments were performed on the test day. After the mice completed the test, these mice were returned to animal cages.

### Reinstatement of Condition Place Preference (day 20)

Following eight days of extinction, mice who had been previously conditioned with cocaine and achieved extinction criterion were primed with a half dose of cocaine (10 mg/kg, i.p.). The reinstatement test was initiated to analyze the effects of Ex4 treatment on the reinstatement of the cocaine-elicited CPP. For the saline-conditioned control group, mice were primed with saline (5 ml/kg, i.p.).

### Locomotor Activity

The general procedure used for locomotor activity was similar to a previously described procedure (Meye et al., 2016). Locomotor activity as total distance that each mice traveled in chambers during the 20 min was recorded while animals were subject to the behavioral testing session above.

### Western Blotting Analysis

After completing the behavioral experiment, mice were euthanized by cervical dislocation, and their brains were immediately removed, flash-frozen in liquid nitrogen, and stored at −80°C. Western blotting was performed to determine the hippocampal expression levels of TLR4, TNF-α, and IL-1β proteins after drug addiction. Hippocampal tissue samples (∼50 mg) were prepared, ground in a homogenizer, and extracted using a BCA Protein Extraction Kit (cat. no. KGP2100; KeyGEN Biotechnology Co., Ltd., Jiangsu, China). Protein samples from each group were then separated by sodium dodecyl sulfate-polyacrylamide gel electrophoresis and transferred to polyvinylidene difluoride membranes (Millipore, United States). The membranes were blocked with 5% skim milk powder for 2 h at room temperature and then incubated in primary antibodies targeting TLR4 (1:1,000; Abcam), TNF-α (1:1,000; Abcam), IL-1β (1:500; Bioworld Technology), and β-actin (1:2,000; Abcam) for more than 24 h at 4°C. Membranes were then washed three times for 5 min each in TBST and incubated with the corresponding secondary antibody (1:1,000) at room temperature for 2 h. Enhanced chemiluminescence reagent was used to detect proteins, and the ratio of the gray value of the target protein band to the gray level of the β-actin band was used to quantify the relative expression level of the target protein. Normalized methods were used for Western blot quantification in order to minimize the impact of variations of experimental error. At great length, the stained blot was imaged, line was drawn around the target protein (TLR4, TNF-α, and IL-1β) or β-actin in each lane, and then the grayscale values were measured using Image J software. The relative value of each of the target protein levels was expressed as the gray values of each target protein divided by the corresponding β-actin. By this means, the relative gray values in different groups was calculated, and then it was divided by the saline group. Ultimately, the normalizing Western blot results of the protein in four groups was procured.

### Immunofluorescence Staining

Following completion of behavioral tests, mice were euthanized by cervical dislocation, and their brains were immediately removed and flash-frozen in ice-cold isopentane. Next, 20-μm-thick slices were collected from the dorsal hippocampus, thaw-mounted on slides, and stored at −80°C until use. Specimens were dehydrated with sucrose, frozen, and then soaked in phosphate-buffered saline solution for 10 min. Tissue sections were then treated with 3% hydrogen peroxide and incubated with 3% bovine serum albumin, followed by incubation with anti-TLR4 antibodies (1:1,000) overnight at 4°C. After washing, the sections were incubated in secondary antibodies at room temperature for 1 h, and nuclei were stained with 4′,6-diamidino-2-phenylindole (Cat. no. ZLI-9557; ZSGB-BIO, Beijing, China). Images were captured with a Leica DM6 fluorescence microscope (Leica, Germany) and the fluorescence density among different groups was compared using Image J, which was similar to the literature (Jensen, 2013).

### Statistical Analysis

GraphPad Prism 8.4.0 (GraphPad Software) and SPSS 23.0 were used for statistical analysis for all experiments. All data are presented as means ± standard errors of the means. The data from cocaine-induced CPP between pre-conditioning and post-conditioning was analyzed using paired t-test. One-way repeated measures analysis of variance (ANOVA) was also used to analyze the statistical significance of effects of Ex4 on cocaine-induced CPP and TLR4, TNF-α, IL-1β proteins in the hippocampus. *Post hoc* analyses of significant effects were conducted using the Tukey’s, Dunnett’s or Bonferroni test. For multiple groups comparison, two-way ANOVA with one repeated measurement was performed between-subjects factors of drug treatment and within-subjects factors of test session. Student’s t-test was used for comparisons between independent groups. Differences with *p* values < 0.05 were considered statistically significant.

## Results

### Effects of Ex4 Pretreatment on the Acquisition of Cocaine-Induced Condition Place Preference

**FIGURE 1:**
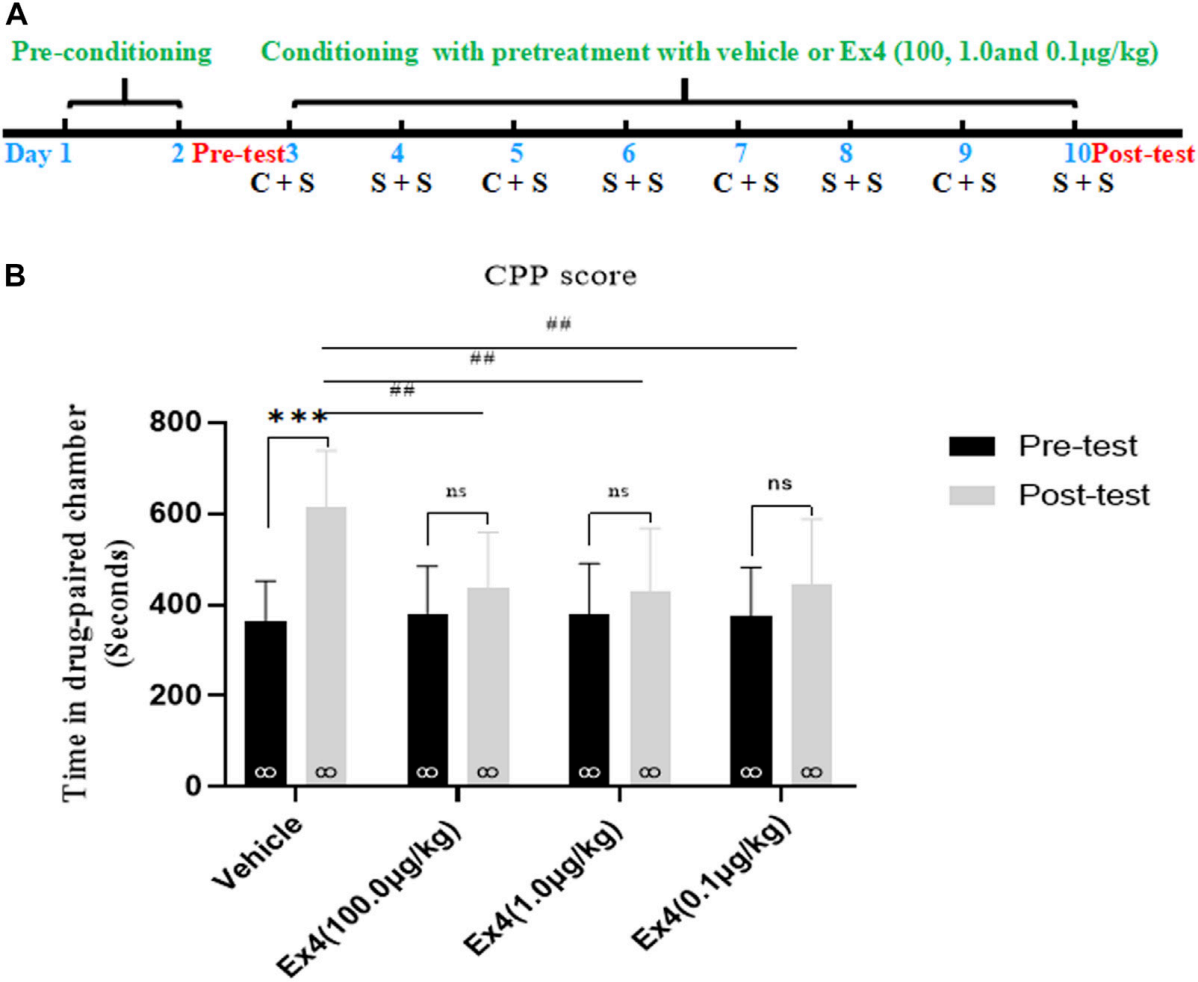
Ex4’s pretreatment prevents the acquisition of cocaine-induced CPP. **(A)** The experimental schedule for saline (S) as well as cocaine (C) and Ex4 treatments. **(B)** CPP scores following systemic treatment with vehicle and Ex4 (100, 1.0, and 0.1 μg/kg) during cocaine conditioning show that cocaine induced a significant CPP that was prevented by Ex4 pretreatment. *** represents *p* < 0.001 vs pretest, ns represents no significant difference, paired t-test. ^##^ represents *p* < 0.01 vs vehicle, one-way ANOVA followed by a Dunnett’s *post hoc* test. All data are presented as the mean ± SEM.

We firstly examined the role of repeated pretreatment of Ex4 (100.0, 1.0, and 0.1 μg/kg) in the acquisition of cocaine-induced CPP. Each mouse was pretreated with saline (5 ml/kg) or Ex4 60 min before i.p. injection of cocaine or saline and was then treated with an i.p. injection of cocaine-HCl (20 mg/kg) or an equal volume of saline (5 ml/kg) during the cocaine conditioning period alternately every other day. The implemention of experimental timeline was described as in Figure 1A. Four out of 36 mice were excluded because they spent less than 240 s or more than 960 s in any chamber in the state of pretest. And then the remaining 32 mice were then randomly assigned to four independent groups: vehicle, Ex4 (100.0 μg/kg), Ex4 (1.0 μg/kg), and Ex4 (0.1 μg/kg) groups, and experienced eight-days CPP training. Vehicle (saline 1 ml/kg) and Ex4 (100.0, 1.0, and 0.1 μg/kg) were administered to animals in the vehicle and Ex4 treatment groups 1 h before injection of cocaine or saline. As shown in Figure 1B, a paired t-test showed that after experiencing 8-days of cocaine CPP training, cocaine induced a significant increase in CPP score in vehicle group compared to the baseline preference (t_7_ = 6.636, *p* < 0.001). However, cocaine failed to produce the similar CPP response in the Ex4 groups at the dose of 10 μg/kg (t_7_ = 1.748, *p* > 0.05), Ex4 1 μg/kg (t_7_ = 1.527, *p* > 0.05), and Ex4 0.1 μg/kg (t_7_ = 1.876, *p* > 0.05). Furthermore, a one-way ANOVA (repeated measures) showed that there was a statistical difference in time spent in the cocaine-paired chamber among these four groups [F_(3,31)_ = 15.12, *p =* 0.0002, Figure 1B], and Dunnett’s multiple comparisons *post hoc* analysis revealed that Ex4 at the dose of 10.0 μg/kg (*p* = 0.0035), 1.0 μg/kg (*p* = 0.0027), and 0.1 μg/kg (*p* = 0.0089) significantly decreased the CPP score in comparison with the vehicle group. Therefore, these results suggest that Ex4 pretreatment prevents the acquisition of cocaine-induced CPP.

### Effects of a Single Injection of Ex4 on the Expression of Cocaine-Induced Condition Place Preference

**FIGURE 2:**
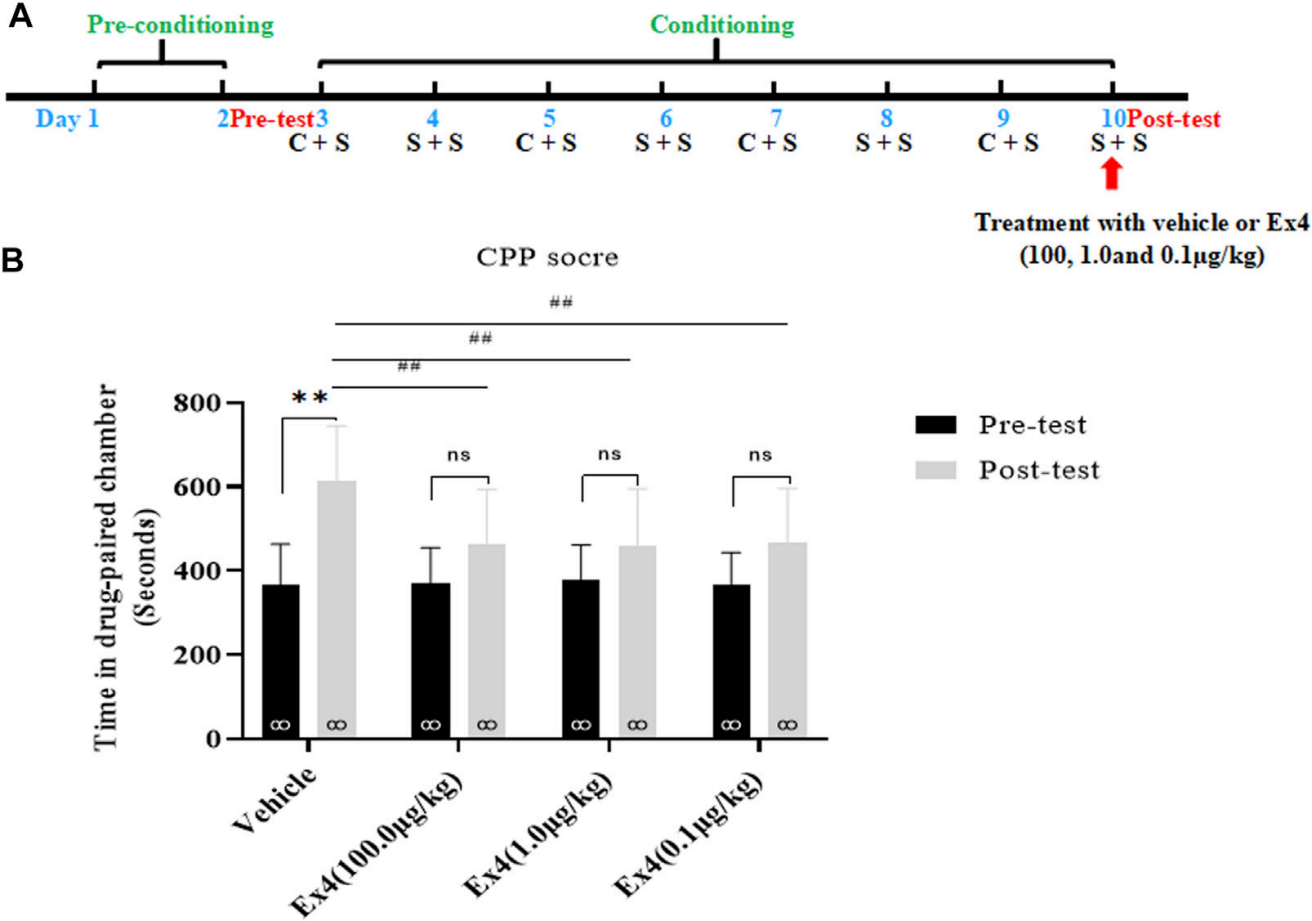
Ex4’s single treatment blocks expression of cocaine-induced CPP. **(A)** The experimental schedule for saline (S) as well as cocaine (C) and Ex4 treatments. **(B)** CPP scores following a single treatment withvehicle and Ex4 (100, 1.0, and 0.1 μg/kg) on the CPP test day show that cocaine induced a significant CPP that was blocked by systemic Ex4 treatment. ** represents *p* < 0.01 vs pretest, ns represents no significant difference, paired t-test. ^##^ represents *p* < 0.01 vs vehicle, one-way ANOVA followed by a Dunnett’s *post hoc* test. All data are presented as the mean ± SEM.

**FIGURE 3:**
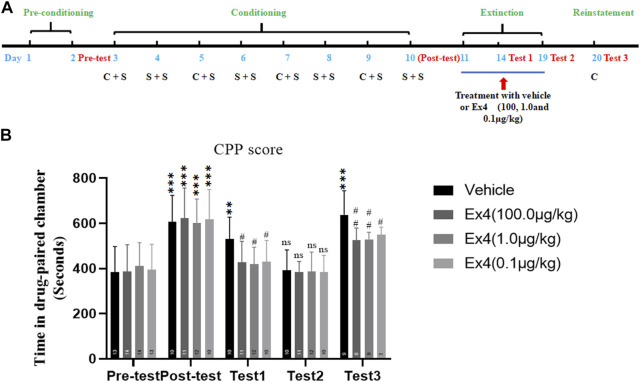
Ex4’s chronic treatment facilitates extinction and inhibits reinstatement of cocaine-induced CPP. **(A)** The experimental schedule for saline (S) as well as cocaine (C) and Ex4 treatments. **(B)** CPP scoresfollowing systemic treatment with vehicle and Ex4 (100, 1.0, and 0.1 μg/kg) during the extinction show thatpost-extinction Ex4 treatment facilitates extinction of CPP and inhibits cocaine-primed reinstatement comparedto the vehicle group. ** and *** represent *p* < 0.01 vs pretest and *p* < 0.01 vs pretest, ns represents no significantdifference, Student’s t test. ^#^ and ^##^ represent *p* < 0.05 vs vehicle and *p* < 0.01 vs vehicle, one-way ANOVA followed by a Dunnett’s *post hoc* test. All data are presented as the mean ± SEM.

We further investigated the impacts of a single infusion of Ex4 on the expression of cocaine-induced CPP. As shown in **Figure 3A**, the general procedure of this experiment protocol was similar to experiment one except that Ex4 was intraperitoneally injected at dose of 100.0, 1.0, and 0.1 μg/kg only on the CPP test day. In total, there are 4 mice were discarded because of original room bias. Subsequently, the remaining animals were separated into four independent groups and exposed to the next 8-days of cocaine-associated CPP training, and CPP test. As shown in Figure 2B, a paired t-test showed that cocaine induced a significant elevation in CPP score in the vehicle group when compared to the initial preference (t_7_ = 4.709, *p* = 0.0022). However, cocaine had no significant effects in the Ex4 groups at the dose of 10 μg/kg (t_7_ = 1.612, *p* > 0.05), Ex4 1 μg/kg (t_7_ = 1.355, *p* > 0.05), and Ex4 0.1 μg/kg (t_7_ = 1.759, *p* > 0.05). Furthermore, a one-way ANOVA (repeated measures) revealed a statistical difference in time spent in the cocaine-paired chamber among these groups [F_(3,31)_ = 16.96, *p =* 0.0013, Figure 2B], and Dunnett’s multiple comparisons *post hoc* analysis showed that Ex4 at the dose of 100.0 μg/kg (*p* = 0.0090), 1.0 μg/kg (*p* = 0.0086) and 0.1 μg/kg (*p* = 0.0046) produced significant reduction in the CPP score in comparison with the vehicle group. Thus, these results indicate that an acute single injection of Ex4 inhibits the expression of cocaine-induced CPP.

### Effects of Repeated Injection of Ex4 on the Extinction and Reinstatement of Cocaine-Induced Condition Place Preference

We assessed the ability of post-extinction Ex4 to modulate the extinction and reinstatement of cocaine-induced CPP. The general experimental flow as well as Ex4 and cocaine treatments were performed as shown in Figure 2A. In total, 21 mice were ruled out from this experiment. Animals were randomly separated into four groups and conditioned to cocaine (20 mg/kg, ip) on alternating days. After completion of 8-days of consecutive CPP training, a room preference for the cocaine-paired chamber was established in all groups when compared with the CPP responses in the preconditioning phase (*p* < 0.001 for vehicle, 100.0, 1.0, and 0.1 μg/kg Ex4 groups, Student’s t-test Figure 3B). Subsequently, mice were daily treated with Ex4 (10.0, 1.0, and 0.1 μg/kg) or vehicle immediately after each extinction session. In test 1, the CPP was incompletely extinguished when compared with the CPP responses in the preconditioning state (*p* = 0.0038 for vehicle group, Student’s t test Figure 3B). Additionally, one-way rmANOVA showed that there was a significant difference among four groups in the test 1 [F_(3,42)_ = 3.547, *p* = 0.0230, Figure 3B], and Dunnett’s multiple comparisons *post hoc* analysis revealed that the CPP score was reduced by Ex4 at dose of 10.0 μg/kg (*p* = 0.0320), 1.0 μg/kg (*p* = 0.0162), and 0.1 μg/kg (*p* = 0.0454) compared to the vehicle group. These results illustrated that repeated administration of Ex4 strengthened the extinction. Different from test 1, the CPP was completely extinguished in test 2 when compared with the CPP responses in the preconditioning stage (*p* > 0.05 for vehicle, 100.0, 1.0, and 0.1 μg/kg Ex4 groups, Student’s t test Figure 3B). Further, the effects of post-extinction Ex4 on the reinstatement of cocaine-CPP was observed. On the reinstatement day, priming with cocaine (10 mg/kg, ip) in the vehicle group produced a reinstatement of the cocaine-induced CPP (t_21_ = 5.294, *p* < 0.001, Student’s t-test). One-way repeated measures ANOVA revealed a statistical difference in the CPP score among vehicle and Ex4 treatment groups [F_(3,32)_ = 5.643, *p* = 0.0036, one-way rmANOVA, Figure 3B], and Dunnett’s multiple comparisons showed that the CPP response was significantly attenuated by the administration of Ex4 at dose of 10.0 μg/kg (*p* = 0.0039), 1.0 μg/kg (*p* = 0.0044), and 0.1 μg/kg (*p* = 0.0348), indicating that Ex4inhibited the reinstatement. Altogether, these results suggest that repeated injection of Ex4 during extinction promotes the extinction and attenuate the reinstatement of cocaine-induced CPP.

### Effects of Single Injection of Ex4 on the Reinstatement of Cocaine-Induced Condition Place Preference

**FIGURE 4:**
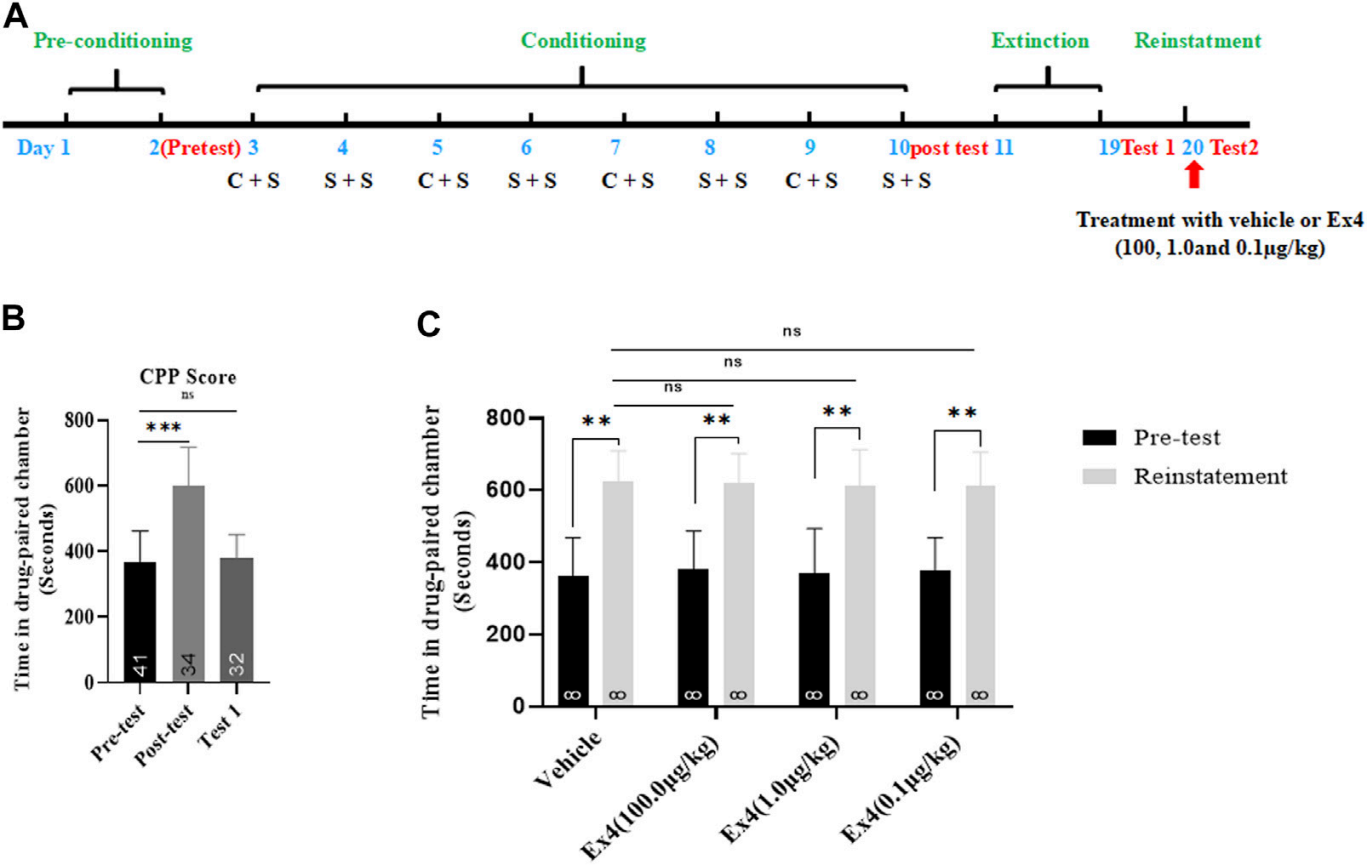
Ex4’s single treatment does not attenuate reinstatement of cocaine-induced CPP. **(A)** The experimental schedule for saline (S) as well as cocaine (C) and Ex4 treatments. **(B)** CPP scores following cocaine conditioning and extinction show that compared to the pretest cocaine treated mice (*n* = 34) induced asignificant CPP, which was extinguished by extinction training. *** represents *p* < 0.001 vs pretest, ns represents no significant difference, Student’s t-test. **(C)** CPP scores following an acute single treatment with vehicle and Ex4 (100, 1.0, and 0.1 μg/kg) on the reinstatement day show that animals treated with Ex4 (*n* = 8) have no significant effects on preventing cocaine-triggered reinstatement compare to vehicle (*n* = 8). ** represents *p* < 0.01 vs pretest, paired t-test. ns represents no significant difference, one-way ANOVA followed by a Dunnett’s *post hoc* test. All data are presented as the mean ± SEM.

We next examined the actions of single administration of Ex4 on the reinstatement of cocaine-induced CPP. The general experimental timeline was shown in Figure 4A. From preconditioning to extinction session, a total of 9 mice were excluded. As shown in Figure 4B, a Student’s t-test showed that the overwhelm majority of animals exhibited a strong place preference for the cocaine-paired chamber after they were subject to the consecutive eight-days of CPP training (t_74_ = 9.65, *p* < 0.001). The extinction standard was achieved because there is no difference in CPP score between the test 1 and the pretest (t_72_ = 0.5814, *p* = 0.5628, Student’s t-test, Figure 4B). Next, these animals were randomly divided into four independent groups and received vehicle or different dose of Ex4 (10.0, 1.0 and 0.1 μg/kg) on the challenge day to investigate the effects of single infusion of Ex4 on the cocaine-primed reinstatement of CPP. As a result, half dose of cocaine produced a significant place preference for the cocaine-paired chamber among all groups regardless of Ex4 treatment (*p* = 0.0033, *p* = 0.0022, *p* = 0.0028 and *p* = 0.0025 for vehicle, 100.0, 1.0 and 0.1 μg/kg Ex4, respectively, Paired t test, Figure 4C). Furthermore, as shown in Figure 4C, one-way rmANOVA showed that there was no significant induction among these groups when compared to the vehicle group [F_(3,31)_ = 0.1448, *p* = 0.8364], and Dunnett’s multiple comparisons *post hoc* analysis also revealed that this CPP response can not be blocked by single administration of Ex4 at dose of 10.0 μg/kg (*p* = 0.9277), 1.0 μg/kg (*p* = 0.9574), and 0.1 μg/kg (*p* = 0.8993). Consequently, these results indicate that single infusion of Ex4 fails to attenuate the reinstatement of cocaine-induced CPP.

### Effects of Ex4 Pretreatment on the Acquisition of the Cocaine-Induced Condition Place Preference and Aberrant Expression of TLR4, TNF-α, and IL1-β in the Hippocampus

**FIGURE 5:**
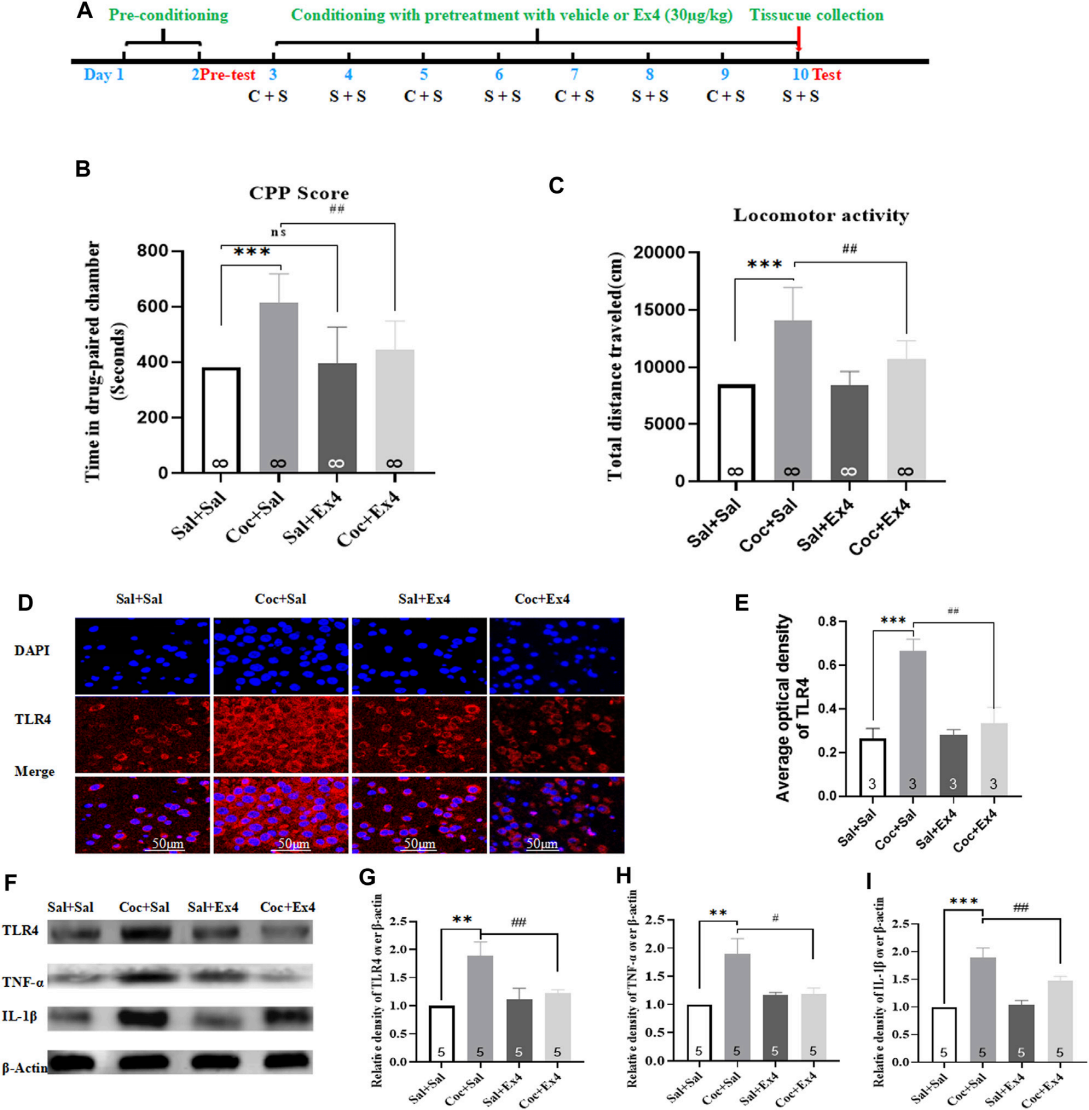
Ex4’s pretreatment inhibits the acquisition of cocaine-induced CPP and alleviates expression levels of TLR4, TNF-α and IL-1β. **(A)** The experimental schedule for saline (S) as well as cocaine (C) and exendin-4 treatment. **(B)** CPP scores following cocaine conditioning with systemic treatment with saline and Ex4 (30.0 μg/kg) show that compared to Sal+Sal group cocaine induced a significant CPP that was lessened by Ex4 treatment. *** represents *p* < 0.001 vs Sal+Sal, ^##^ represent *p* < 0.01 vs Coc+Sal, ns represents no significant difference, one-way ANOVA followed by a Bonferroni’s *post hoc* test. **(C)** Locomotor activity following cocaine conditioning with systemic pretreatment with saline and Ex4 (30.0 μg/kg) show that a hyperactivity induced by cocaine was reduced by Ex4 treatment. *** represents *p* < 0.001 vs Sal+Sal , ^##^ represent *p* < 0.01 vs Coc+Sal, one-way ANOVA followed by a Bonferroni’s *post hoc* test. **(D)** Representative images of immunofluorescent staining with TLR4 (red), and DAPI (blue) in the hippocampus from the different groups (scale bar = 50 μm). Systemic administration of Ex4 diminished fluorescence density of TLR4 up-regulated by cocaine exposure (*n* = 3 in each group). **(E)** Semiquantitative analysis of the relative density of TLR4 by densitometric analysis in different groups. *** represents *p* < 0.001 vs Sal+Sal, ^##^ represent *p* < 0.01 vs Coc+Sal, one-way ANOVA followed by a Tukey’s *post hoc* test. **(F)** Representative immunoblots of TLR4, TNF-α and IL-1β proteins in different groups (*n* = 5 in each group). **(G)** Semiquantitative analysis of the relative levels of TLR4 by densitometric analysis in different groups. *** represents *p* < 0.001 vs Sal+Sal , ^##^ represent *p* < 0.01 vs Coc+Sal, one-way ANOVA followed by a Tukey’s *post hoc* test. **(H)** Semiquantitativeanalysis of the relative levels of TNF-α by densitometric analysis in different groups. ** represents *p* < 0.01 vs Sal+Sal, ^#^ represent *p* < 0.05 vs Coc+Sal, one-way ANOVA followed by a Tukey’s *post hoc* test. **(I)** Semiquantitative analysis of the relative levels of IL-1β by densitometric analysis in different groups. *** represents *p* < 0.001 vs Sal+Sal, ^##^ represent *p* < 0.01 vs Coc+Sal, one-way ANOVA followed by a Tukey’s posthoc test. All data are presented as the mean ± SEM. Sal indicates saline. Coc indicates cocaine. Ex4 indicates Exendin-4.

The experimental schedule for Ex4 and cocaine treatments was described in Figure 5A. Six mice were ruled out from the present experiment. At the end of the pretest, mice were randomly divided into four groups including Sal + Sal (pretreatment with saline injection 1 h prior to saline administration), Coc + Sal (pretreatment with an equal volume of saline 1 h prior to cocaine administration), Sal + Ex4 (pretreatment with Ex4 1 h prior to saline administration), and Coc + Ex4 (pretreatment with the same dose of Ex4 as for the Sal + Ex4 group 1 h before cocaine administration), and then underwent the following eight days of cocaine-CPP training. As shown in Figure 5B, one-way rmANOVA showed that there was significant difference among these groups after a cocaine CPP training [F_(3,31)_ = 8.990, *p* = 0.0062]. In addition, Bonferroni *post hoc* analysis also revealed that cocaine produced a significant CPP compared to the Sal + Sal group (*p* < 0.001), and that this CPP response was attenuated by Ex4 pretreatment (*p* = 0.0039) compared to the Coc + Sal group. As shown by Bonferroni *post hoc* analysis, the CPP score in Ex4+Sal group does not differ from the Sal + Sal group (*p >* 0.05), suggesting that Ex4 treatment alone during CPP conditioning did not produce CPP paradigm compared to the Sal + Sal group. Similar to CPP response, Ex4 pretreatment had a statistically significant effect on the hyperactivity elicited by cocaine was observed [F_(3,31)_ = 16.44, *p* = 0.0008, Figure 5C], and Bonferroni *post hoc* analysis indicated that cocaine increased the locomotion compared to the Sal + Sal group (*p* = 0.0004, Figure 5C) that was reduced by Ex4 compared to the Coc + Sal group (*p* = 0.0079). Overall, these findings showed that the strong CPP response and cocaine conditioned locomotion were attenuated by Ex4 pretreatment.

After the completion of test, all animals were sacrificed and the hippocampus tissues was harvested to detect the expressions levels of TLR4, TNF-α, and IL1-β using Western blot and immunofluorescence staining. The reason why we chose hippocampus is that it has been considered the center of the learning of associations between the environmental context and unconditioned stimuli in mammals (Bohbot and Corkin, 2007; Kim and Fanselow, 1992). Immunofluorescence staining demonstrated that neuronal lesions caused by cocaine re-exposure in the hippocampus were repaired by Ex4 treatment (Figures 5D,E). As depicted in Figure 5E, TLR4 was presented in the cytoplasm and repeated administration of cocaine produced different fluorescence density of TLR4 [F_(3,11)_ = 254.0, *p* = 0.0032, one-way ANOVA], and Tukey’s multiple comparisons test showed that higher density of TLR4 caused by cocaine was significantly reversed by Ex4 treatment (*p* = 0.0031). In line with the results of immunofluorescence staining, Western blotting also showed that there was a statistical difference in the relative expression of TLR4 among these groups [F_(3,19)_ = 42.27, *p* = 0.0002, one-way ANOVA, Figures 5F,G], and Tukey’s *post hoc* analysis revealed that the protein overexpression produced by cocaine was significantly decreased by Ex4 (*p* = 0.0097). Activation of TLR4 facilitates the release of pro-inflammatory cytokine such as IL-1β and TNFα (Brown et al., 2011). Similar to the results of TLR4, Tukey’s multiple comparisons *post hoc* analysis indicated that cocaine produced the higher protein levels of TNF-α (*p* = 0.0057, one-way ANOVA, Figure 5H) and IL1-β (*p* = 0.0009, one-way ANOVA, Figure 5I). Interestingly, compared to the Coc + Sal group, Ex4 significantly alleviated the expression levels of TNF-α (*p* = 0.0326, one-way ANOVA followed by Tukey’s multiple comparisons test, Figure 5H) and IL1-β (*p* = 0.0233, one-way rmANOVA followed by Tukey’s multiple comparisons test; Figure 5I). Taken together, these results revealed that Ex4 pretreatment suppressed the indices of inflammation (TLR4, TNF-α, and IL1-β) within the hippocampus.

### Effects of Ex4 on the Reinstatement of the Cocaine-Induced Condition Place Preference, Locomotor Activity, and Aberrant Expression of TLR4, TNF-α, and IL1-β

**FIGURE 6:**
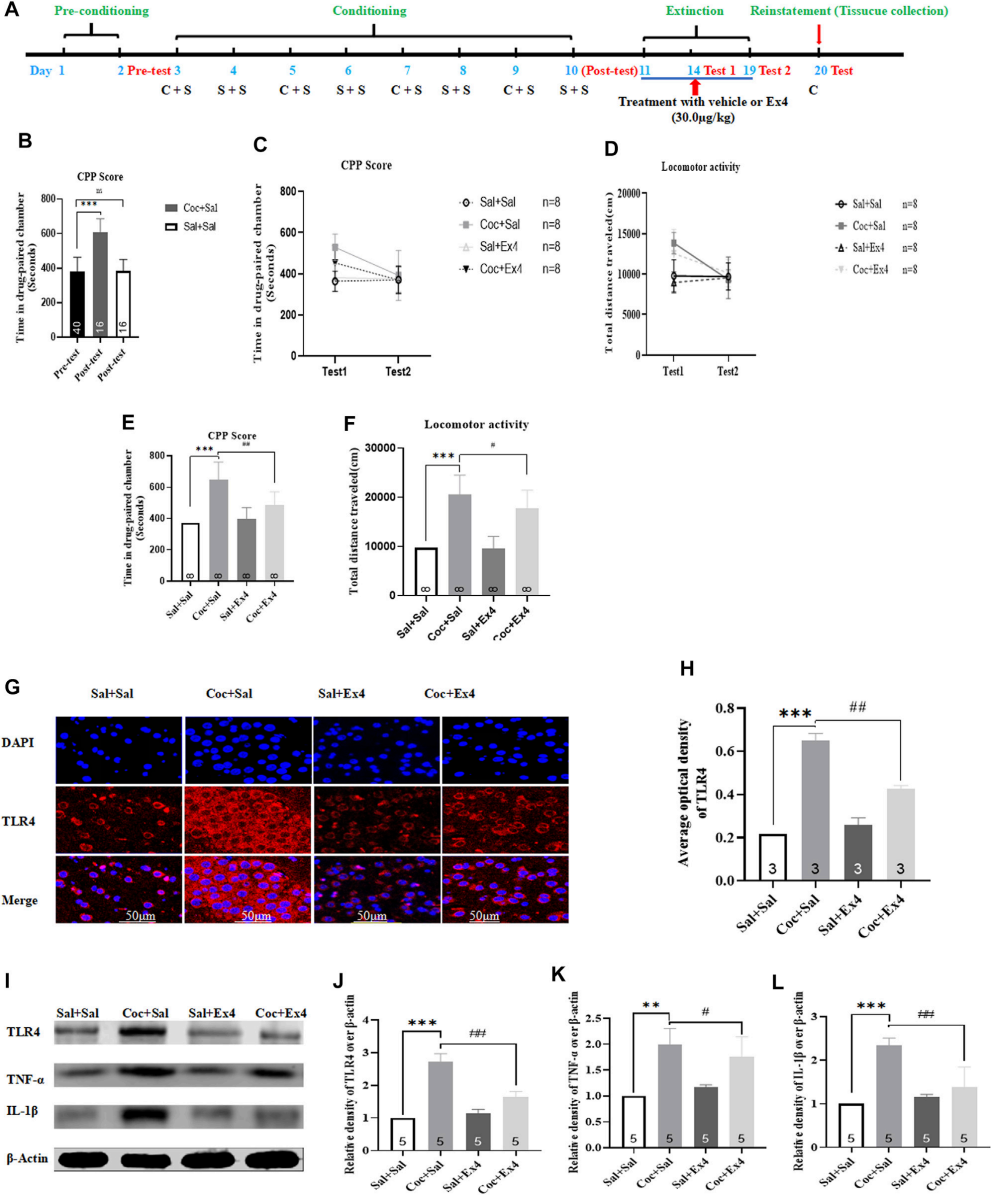
Ex4’s chronic treatment promotes extinction as well as suppresses reinstatement of cocaine-induced CPP and relieves expression levels of TLR4, TNF-α and IL-1β. **(A)** The complete experimental schedule for the behavioral procedure as well as saline (S), cocaine (C), and Ex4 treatment protocols. **(B)** CPP scores of the pre-test and post-test show that cocaine produced a significant CPP response after cocaine conditioning training. *** represents *p* < 0.001 vs pretest, Student’s t test. **(C)** CPP scores following post-extinction Ex4 treatment vehicle and Ex4 (30 μg/kg) immediately after each extinction training show that Ex4 treated animals potentiates extinction of CPP compared to saline treated animals. N = 8 per group, *** represents *p* < 0.001 vs Sal+Sal, ns represents no significant difference, two-way ANOVA followed by a Bonferroni’s *post hoc* test. **(D)** Locomotor activity following post-extinction treatment with saline and Ex4 (30.0 μg/kg) show that Ex4 treated animals reduces locomotion compared to saline treated animals. N = 8 per group, *** represents *p* < 0.001 vs Sal+Sal, ns represents no significant difference, two-way ANOVA followed by aBonferroni’s *post hoc* test. (E) CPP scores after priming with half dose of cocaine show that Ex4 treated animals inhibits cocaine-primed reinstatement compared to saline treated animals. N = 8 per group, *** represents *p* < 0.001 vs Sal+Sal , ^##^ represent *p* < 0.01 vs Coc+Sal, one-way ANOVA followed by a Bonferroni’s *post hoc* test. **(F)** The results of locomotor activity show that Ex4 treatment inhibits cocaine-induced hyperactivity. N = 8 pergroup, *** represents *p* < 0.001 vs Sal+Sal, ^#^ represent *p* < 0.05 vs Coc+Sal, one-way ANOVA followed by a Bonferroni’s *post hoc* test. **(G)** Representative images of immunofluorescent staining with TLR4 (red), andDAPI (blue) in the hippocampus from the different groups (scale bar = 50 μm). Post-extinction Ex4 diminishesfluorescence density of TLR4 up-regulated by cocaine priming (*n* = 3 in each group). **(H)** Semiquantitativeanalysis of the relative density of TLR4 by densitometric analysis in different groups. *** represents *p* < 0.001 vs Sal+Sal, ^##^ represent *p* < 0.01 vs Coc+Sal, one-way ANOVA followed by a Tukey’s *post hoc* test. **(I)** Representative immunoblots of TLR4, TNF-α and IL-1β proteins in different groups (*n* = 5 in each group). **(J)** Semiquantitative analysis of the relative levels of TLR4 by densitometric analysis in different groups. *** represents *p* < 0.001 vs Sal+Sal, ^##^ represent *p* < 0.01 vs Coc+Sal, one-way ANOVA followed by a Tukey’s *post hoc* test. **(K)** Semiquantitative analysis of the relative levels of TNF-α by densitometric analysis in differentgroups. ** represents *p* < 0.01 vs Sal+Sal, # represent *p* < 0.05 vs Coc+Sal, one-way ANOVA followed by a Tukey’s *post hoc* test. **(L)** Semiquantitative analysis of the relative levels of IL-1β by densitometric analysis indifferent groups. *** represents *p* < 0.001 vs Sal+Sal, ^##^ represent *p* < 0.01 vs Coc+Sal, one-way ANOVA followed by a Tukey’s *post hoc* test. All data are presented as the mean ± SEM. Sal indicates saline. Cocindicates cocaine. Ex4 indicates Exendin-4.

The timeline was depicted in Figure 6A. There are a total of eight animals were excluded because of CPP training failure. Afterward, the remaining 32 mice were randomly divided into two groups including Sal + Sal group (administrated with saline alone during conditioning) and Coc + Sal group (administrated with saline and Coc during conditioning). As shown in Figure 6B, most animals spent more time in the cocaine-paired chamber after cocaine CPP training (t_55_ = 9.631, *p* < 0.001, Student’s t-test), revealing that mice treated with repetitive administration of cocaine (20 mg/kg) induced a significant CPP score (609.3 ± 92.3 s) when compared to the CPP responses in the pretest (381.6 ± 51.2 s). However, repeated administration of saline failed to induce a CPP (t_54_ = 0.067, *p* > 0.05, Student’s t-test). Subsequently, all mice were subject to Ex4 or saline treatments daily administrated immediately after each extinction session and divided into four groups including Sal + Sal group, Coc + Sal group, Sal + Ex4 group and Coc + Ex4 group to assess the effects of Ex4 on the extinction and reinstatement of cocaine-induced CPP. The CPP response of the mice showed an significant difference among these groups [treatment: F_(1, 28)_ = 7.933, *p* = 0.0088; test: F_(3,28)_ = 5.299, *p* = 0.0051; interaction F_(3,28)_ = 3.032, *p* = 0.0458; two-way rmANOVA, Figure 6C], and Bonferroni’s multiple comparisons test indicated that the CPP score of the Coc + Sal group remains higher than the Sal + Sal group (*p* = 0.003). On one hand, these results suggested that administration of Ex4 had a significant effect on the extinction of CPP. On the other hand, these results also revealed that the CPP response was not completely extinguished in test 1. As shown in Figure 6C, however, on test 2, two-way rmANOVA followed by Bonferroni’s multiple comparisons test revealed that animals among these groups had no difference in CPP score when compared to the Sal + Sal group (*p* > 0.05), indicating that they achieved extinction criteria. Similarly, there is a statistical difference in locomotor activity among there groups [treatment: F_(1, 28)_ = 8.904, *p* = 0.0099; test: F_(3,28)_ = 6.127, *p* = 0.0046; interaction F_(3,28)_ = 6.265, *p* = 0.0013; two-way rmANOVA, Figure 6D]. Subsequently, we observed the effects of chronic Ex4 treatment on the cocaine-induced reinstatement of CPP. On the reinstatement test day, one-way rmANOVA showed that there was significant difference in CPP score among these groups after priming with half dose of cocaine [F_(3,31)_ = 21.56, *p* < 0.001, Figure 6E], and Bonferroni *post hoc* analysis revealed that compared to the Sal + Sal group cocaine produced a significant CPP (*p* = 0.0008) that was attenuated by Ex4 treatment (*p* = 0.0081). Additionally, one-way rmANOVA also showed that there was significant difference among these groups after priming with half dose of cocaine [F_(3,31)_ = 42.01, *p* < 0.001, Figure 6F], and Bonferroni *post hoc* analysis revealed that compared to the Sal + Sal group cocaine significantly increased locomotor activity (*p* = 0.0009) that was attenuated by Ex4 treatment (*p* = 0.0426). Overall, these results indicate that post-extinction Ex4 does not only inhibit the reinstatement of cocaine-CPP but also reduced the locomotion elicited by priming with cocaine on the reinstatement day.

Following the reinstatement test, the molecular mechanisms of these therapeutic actions of Ex4 were evaluated using Western blotting and immunofluorescence staining. As shown in Figures 6G,H, the Sal + Sal, Coc + Sal group, Sal + Ex4 and Coc + Ex4 groups displayed different fluorescence intensity [F_(3,11)_ = 421.3, *p* = 0.0002, one-way ANOVA], and Tukey’s multiple comparisons *post hoc* analysis showed that the fluorescence intensity of TLR4 which was enhanced by cocaine (*p* = 0.0003) was significantly attenuated by Ex4 (*p* = 0.0063). Similar to result of immunofluorescence staining, Western blotting also revealed the different protein content of TLR4 among these groups [F_(3,19)_ = 132.1, *p* < 0.0001, one-way ANOVA, Figures 6I,J), and Tukey’s multiple comparisons *post hoc* analysis showed that cocaine increased the protein level of TLR4 (*p* = 0.0004) that was significantly reduced via Ex4 treatment (*p* = 0.0077). Consistent with these results, Tukey’s multiple comparisons test showed that the levels of TNF-α (*p* = 0.0064, one-way ANOVA, Figure 6K) and IL-1β (*p* = 0.0002, one-way ANOVA, Figure 6L) were significantly higher in Coc + Sal group than in Coc + Ex4 group. Similarly, Tukey’s multiple comparisons *post hoc* analysis showed that treatment with Ex4 significantly relieved the expression levels of TNF-α (*p* = 0.0351, one-way ANOVA, Figure 6K) and IL1-β (*p* = 0.0078, one-way ANOVA, Figure 6L). In summary, these results indicates that Ex4 alleviates TLR4-associated inflammatory signaling.

## Discussion

In the current study, we demonstrated that the different dose of GLP-1R analog Ex4 (administrated daily during the cocaine conditioning) inhibited the acquisition and expression of cocaine-induced CPP. Moreover, we showed that repetitive Ex4 treatment (immediately after the each extinction session) did not only facilitate the extinction of cocaine-associated CPP but also attenuated reinstatement elicited by a single infusion of cocaine (10 mg/kg on the reinstatement day). Finally, we found the molecular mechanisms underlying these therapeutic effects of Ex4 on ameliorating these cocaine-mediated behaviors were closely associated with down-regulation of inflammatory cytokines in the hippocampus, including TLR4, TNF-α, and IL-1β. Consequently, these findings provide additional support for the potential role of glucagon-like peptide-1 agonist in the cocaine-mediated behavior amelioration.

The condition place preference (CPP) paradigm, based on classical Pavlovian conditioning, has traditionally been considered to be one of the most commonly behavioral models applied to study the rewarding properties of psychostimulants (Tzschentke, 1998; Bardo and Bevins, 2000). In the CPP paradigm, the addictive drug’s rewarding effects functions as an unconditioned stimuli (United States), whereas the conditioned stimuli (CS) actions as a secondary stimuli which was acquired mainly by repeatedly pairing the rewarding properties of drugs with environmental stimuli in the process of cocaine conditioning. In line with previous work (Meye et al., 2016; Ladrón de Guevara-Mirand et al., 2018), we also found that systemic administration of cocaine (20 mg/kg) during conditioning phase is competent to produce a significant CPP paradigm for the overwhelming majority of animals. These indicate that although no cocaine was available on the CPP test day, the familiar drug-paired compartment was still preferred by mice. Further, these results also illustrate that the rewarding efficacy of cocaine well paired with the designed drug-paired compartment. Different from cocaine, however, Ex4 and saline were inadequate to induce a room preference or aversion, identical to previous work demonstrating that Ex4 itself did not produce a CPP (Egecioglu et al., 2013). Importantly, these cocaine-induced behavioral changes including CPP learned by mice in the previous drug-paired environment could be abolished by systemic Ex4 administration (Skibicka, 2013; Engel and Jerlhag, 2014; Hayes and Schmidt, 2016), fitting with the findings in our study demonstrating that single or repeated pretreatment with Ex4 significantly produced a significant reduction in the time spent in the cocaine-affiliated chamber on the CPP test day. Overall, repeated or single administration of Ex4 have a profound influence on the amelioration of addictive cocaine-mediated behaviors such as the acquisition and expression of CPP paradigm.

Extinction training is an active learning process that leads to a gradual decline in acquired response. It generates the new learning and memories that predict no more delivery of addictive drugs, through which the expression of initial memories of addictive drugs to control behavior was suppressed (Marlatt, 1990; Chesworth and Corbit, 2015), and thereby reducing the susceptibility of relapse (Heather and Bradley, 1990). However, extinction exposure alone proves insufficient (Marlatt, 1990). Additional studies have determined that extinction can be clearly promoted by pharmacological treatment (Botreau et al., 2006; Paolone et al., 2009). However, to our knowledge, few studies have investigated the role of Ex4 in extinction of cocaine-related behavior to date. Thus, elucidating the potential effects of Ex4 on extinction of cocaine-induced CPP is another important purpose in the present study. As reported previously (Botreau et al., 2006; Paolone et al., 2009), our work found that mice treated with Ex4 appear to extinguish more quickly than mice treated with saline in the first three days of extinction training. Additionally, our findings further imply that the new learning and memories induced by combination between Ex4 and extinction training significantly suppress the initial memories of cocaine-related CPP, thereby enhancing the extinction.

The high incidence of relapse after abstinence is the primary hallmark of cocaine addiction (Leshner, 1997). However, the effects of available medication and treatment strategies on relapse is limited (O’Brien, 1997). Manipulating memory reconsolidation reportedly proposed as another more effective treatment option to decrease the relapse of cocaine addiction (Rich and Torregrossa, 2018). Using reinstatement paradigm model that has been viewed as the most commonly used animal model of relapse of cocaine-associated behavior (Schmidt et al., 2005), the therapeutic actions of Ex4 treatment on the reconsolidation and reinstatement of cocaine-related behavior were evaluated in the present study. We found that a significant CPP was acquired after a period of extinction and priming injection of a half dose of cocaine, indicating that simultaneous re-exposure to the context cues previously associated with cocaine abuse and cocaine itself precipitated a craving-induced relapse, and that the drug-triggered reinstatement paradigm has been successfully established as well. In addition, an acute single injection of cocaine (on the reinstatement day) markedly increased locomotion that was clearly affected by post-extinction Ex4. Similarly, in our work, post-extinction Ex4 treatment with combination of the extinction training received desirable actions on attenuating reinstatement of CPP through affecting the memory reconsolidation. However, single administration of Ex4 on reinstatement day failed to produce similar effects on extinction and reinstatement. The precise reason why single injection of Ex4 was unable to block relapse remain elusive. But the possible explanation is that single injection of Ex4, lack of combination of extinction training, was not sufficient to modulate memories reconsolidation. Consequently, the therapeutic efficacy of Ex4 on reinstatement and memory reconsolidation depends greatly on combination with extinction training. Regardless of the mechanisms underlying the inability of single administration of Ex4 on reinstatement of cocaine-CPP, our results are sufficient to illustrate that Ex4 exert far-reach effects on extinction and reinstatement of cocaine-mediated CPP.

Taken together, the CPP as well as extinction and reinstatement induced by cocaine were significantly affected using systemic administration of Ex4. These findings expanded on these studies showing that the important effects of systemic dose of GLP-1 receptor agonists on addiction-like behaviors in preclinical model of cocaine use disorder (Graham et al., 2013; Hernandez et al., 2018). However, little is known about the underlying mechanisms through which Ex4 ameliorates addictive behaviors associated with cocaine consumption.

### Inflammation and Cocaine Addiction

A variety of work reveal an important role of neuroinflammation in many central nervous system (CNS) diseases (Block and Hong, 2005; Tansey et al., 2007; Chen et al., 2016) and that neuroinflammation was believed to contribute to the progression and maintenance of substance use disorders including cocaine (Hutchinson and Watkins, 2014; Northcutt et al., 2015; Lacagnina et al., 2016). The aberrant regulation of neuroimmune signaling caused by drug consumption may impair neuronal function, aggravate neurodegeneration, and promote neurotoxicity, which might result in drug-associated behaviors via activation of microglia (Lacagnina et al., 2016; Liu et al., 2016; Pocock and Kettenmann, 2007). Furthermore, the microglia activation also promoted the release of pro-inflammatory cytokines including IL-1β and TNF-α, and the generation of reactive oxygen and nitrogen species that cause further neuronal lesion (Beardsley and Hauser, 2014). In line with these data, we also observed that animals who acquired a strong CPP always showed the corresponding over-expression of TLR4, IL-1β, and TNF-α protein in the hippocampus. Further, these animals also showed a higher fluorescence density of TLR4 in the CA3 region of the hippocampus. Thus, it is conceivable that there the over-expression of TLR4, IL-1β, and TNF-α in the hippocampus is strongly associated with cocaine-related behavior, and suppressing these neuroinflammation may serve as a promising treatments for substance use disorders (Kohno et al., 2019).

### Mechanisms Through Which GLP-1R Agonists Attenuate Cocaine Reward

Indeed, several potential anti-inflammatory pharmacotherapies with an ability to inhibit expression of IL-1β and TNF-α, such as Ibudilast (Ray et al., 2014) and Minocycline (Garrido-Mesa et al., 2013) have been shown to exert therapeutic actions on treatments for substance use disorders. Similarly, a recent clinical study showed that the anti-inflammatory agent naloxone, which targets IL-1β and TNF-α, can effectively attenuate dopamine release and thereby prevent the development of CPP in rats (Northcutt et al., 2015). We therefore hypothesized that anti-inflammatory components with the ability to suppress TLR4, IL-1β, and TNF-α signaling may repurposed as modulator of the dopamine release and cocaine-seeking behaviors (Correia et al., 2020). Intriguingly, accumulating studies have demonstrated that GLP-1R agonists Ex4 also possessed the anti-inflammatory properties (Hattori et al., 2010; Ajay et al., 2011; Campbell and Drucker, 2013; Lee and Jun 2016; Pinho-Ribeiro et al., 2017; Thangavel et al., 2017; Géa et al., 2020). For instance, TLR4 (Ajay et al., 2011), TNF-α and IL-1β (Pinho-Ribeiro et al., 2017; Thangavel et al., 2017) were effectively alleviated by Ex4 treatment. Our findings extend these data and identify that Ex4 did not only exert a significantly suppressive effects on the expression TLR4, TNF-α, and IL-1β in hippocampus but also ameliorated the cocaine-related maladaptive behaviors. In addition, we also found that Ex4 repaired neuronal damage caused by cocaine. Collectedly, Ex4 did not only alleviate the expression levels of TLR4, TNF-α, and IL-1β within hippocampus but also repaired the neuronal damage, thereby functioning as a novel anti-inflammatory agents.

Although we have shown that Ex4 ameliorates cocaine-mediated behavior via inhibiting the abnormal expression of TLR4, TNF-α and IL-1β in hippocampus, further studies are needed to investigate the possible direct targets of Ex4. Indeed, recent study have indicated that systemic administration of Ex4 is also distributed in other area of brain such as the accumbens core and shell, Ventral tegmental area (VTA) and Lateral septum (LS) (Hernandez and Schmidt, 2019). Additionally, these nucleus were reportedly associated with the rewarding effects of psychostimulant abuse (Schmidt and Pierce, 2010; Hernandez and Schmidt, 2019). For instance, previous study has demonstrated that peripheral administration of Ex4 inhibit the neurotransmitter dopamine release in the nucleus accumbens after cocaine exposure (Egecioglu et al., 2013; Sorensen et al., 2015). Accordingly, it is conceivable that the therapeutic actions of Ex4 on cocaine-related behaviors may be closely associated with other region of brain including the nucleus accumbens and inhibition of the dopamine release. In the further study, we intend to examine the effects of direct injection of Ex4 into the nucleus accumbens on the dopamine release and the potential mechanism. In addition, it is still unknown whether the over-expression of TNF-α, and IL-1β was directly attributable to the TLR4 activation or chronic cocaine exposure. Further studies are needed to assess whether Ex4 can exert neuroprotective effects for treating cocaine use disorder by blocking TLR3 and TLR2 signaling or through other factors. Moreover, sex differences in the efficacy of Ex4 for reducing cocaine-seeking behaviors were not explored in the current study, and additional research is needed to evaluate whether other anti-inflammatory agents have the same biological effects as Ex4 on other commonly abused drugs. Finally, although the molecular mechanisms of Ex4 were discussed, behavioral and pharmacological treatments should be further investigated in human clinical trials. In conclusion, we found that repeated administration of cocaine significantly augmented the time spent in cocaine-paired chamber, increased locomotor activity, and promoted the release of TLR4 and pro-inflammatory cytokines (IL-1β and TNF-α) in the hippocampus. Notably, most of these alterations caused by chronic cocaine exposure can be significantly blocked by Ex4 treatment through a mechanism involving reduced hippocampal TLR4, IL-1β and TNF-α signaling. On one hand, elucidating the mechanistic role of TLR4, IL-1β and TNF-α in cocaine-induced behavior is essential to ameliorate the understanding of progression and maintenance of cocaine addiction. On another hand, these findings offer innovative insights into the therapeutic effects of the widely available Ex4 on TLR4-associated neuroiflammation in an animal model of cocaine addiction. Therefore, GLP-1R agonist Ex4 might be a promising medication candidate for substance use disorder.

## Data Availability

The original contributions presented in the study are included in the article, further inquiries can be directed to the corresponding authors.
